# A common molecular signature in ASD gene expression: following Root 66 to autism

**DOI:** 10.1038/tp.2015.112

**Published:** 2016-01-05

**Authors:** L Diaz-Beltran, F J Esteban, D P Wall

**Affiliations:** 1 Division of Systems Medicine, Department of Pediatrics, School of Medicine, Stanford University, Stanford, CA, USA; 2 Division of Systems Medicine, Department of Psychiatry, Stanford University, Stanford, CA, USA; 3 Department of Experimental Biology, Experimental Sciences Faculty, University of Jaen, Jaen, Spain; 4 Department of Biomedical Data Science, Stanford University, Stanford, CA, USA

## Abstract

Several gene expression experiments on autism spectrum disorders have been conducted using both blood and brain tissue. Individually, these studies have advanced our understanding of the molecular systems involved in the molecular pathology of autism and have formed the bases of ongoing work to build autism biomarkers. In this study, we conducted an integrated systems biology analysis of 9 independent gene expression experiments covering 657 autism, 9 mental retardation and developmental delay and 566 control samples to determine if a common signature exists and to test whether regulatory patterns in the brain relevant to autism can also be detected in blood. We constructed a matrix of differentially expressed genes from these experiments and used a Jaccard coefficient to create a gene-based phylogeny, validated by bootstrap. As expected, experiments and tissue types clustered together with high statistical confidence. However, we discovered a statistically significant subgrouping of 3 blood and 2 brain data sets from 3 different experiments rooted by a highly correlated regulatory pattern of 66 genes. This Root 66 appeared to be non-random and of potential etiologic relevance to autism, given their enriched roles in neurological processes key for normal brain growth and function, learning and memory, neurodegeneration, social behavior and cognition. Our results suggest that there is a detectable autism signature in the blood that may be a molecular echo of autism-related dysregulation in the brain.

## Introduction

Autism is regarded as one condition among a genetically heterogeneous group of neurodevelopmental syndromes with high prevalence^1^ that has a wide range of phenotypes, collectively grouped together as autism spectrum disorder (ASD). The unifying clinical features across the spectrum involve fundamental impairments in social interaction, communication deficits and highly restrictive interest and/or repetitive behaviors.^2, 3^ Although there is no unifying hypothesis about the molecular pathology of autism, it is clear that the disorder is highly heritable and results from the combination of genetic, neurologic, immunologic and environmental factors. However, it remains unclear whether its genetic component stems from the combination of a few common variants or of many rare variants.^4, 5^

Recent advances in genetics, genomics, developmental neurobiology and systems biology have offered important insights into the molecular agents and biological mechanisms responsible for ASD. Microarray technologies and next-generation sequencing have enabled high-throughput discovery of genes likely to be involved in the molecular pathology of autism.^5, 6, 7, 8^ However, as the success in discovery has risen, the number of candidate genes with associated risk for ASD has also stretched well into the hundreds.^9, 10^ As of December 2014, 667 genes have been implicated in autism (https://gene.sfari.org/autdb/HG_Home.do). Despite the large amounts of data now available, the general lack of replication across studies suggests that more data will be needed to fully characterize the genetic models responsible for the various forms of autism.

These high-throughput and large-scale efforts have confirmed that autism is a multisystem and heterogeneous condition. Thus, understanding the complex genetic architecture of ASD must involve, among other things, the study of autism gene expression across different tissues using integrative approaches. The majority of gene expression experiments conducted so far have been on blood-derived cells and to a lesser extent postmortem brain tissue from autism cases and matched controls. More recent approaches have examined regulatory patterns in induced pluripotent stem cells forming neurons from individuals with autism. Individually, these studies have advanced our understanding of molecular systems involved in either the cause or effect of autism. We propose and test here the notion that together these experiments may help refine our understanding of genes and pathways important in onset and maintenance of autism. Specifically, we perform an integrated systems biology analysis of all published autism gene expression studies to test whether a common signature representative of ASD exists and ultimately if it can be detected in both blood and brain.

## Materials and methods

### Experiments and gene lists

To compile a complete set of published and publically available gene expression experiments we used Nextbio,^11^ an ontology-based platform that provides global collections of high-throughput public data that meet four criteria: broad coverage of genes, existence of a control group, access to raw or normalized data and supply of sample annotations. We downloaded gene expression data and derived lists of differentially expressed genes from 27 case–control biosets of 9 independent experiments covering 657 autism, 9 mental retardation and developmental delay, 566 control samples: GSE37772, GSE25507, GSE7329, GSE28475, GSE38322, GSE6575, GSE18123, GSE28521 and GSE39447.^7, 12, 13, 14, 15, 16, 17, 18, 19^ Nextbio employed Welch or Standard *t*-test, paired and unpaired, as appropriate, to statistically analyze these case–control experiments and established a nominal, unadjusted *P*-value significance cutoff of 0.05 and a minimum absolute fold-change cutoff of 1.2 (the lowest sensitivity threshold of commercial microarray platforms) to select the differentially expressed genes for each bioset. The statistical threshold method used by Nextbio for gene selection aimed to maintain the balance between sensitivity and specificity across all experiments; these cutoffs were deliberately less stringent to guarantee the inclusion of all potentially interesting genes, and have been commonly used for this purpose in the literature.^20, 21, 22, 23, 24, 25, 26^ We then filtered each list of differentially expressed genes obtained from Nextbio to save just the unique gene symbol identities assigned from The Hugo Gene Nomenclature Comittee.^27^ A description and content of each bioset can be found in Supplementary Table S1a. It is important to clarify that, although important covariates exist across these experiments, age and gender were well-matched across a majority of the samples, with most of the mixed-gender biosets having roughly four males to one female (consistent with the male bias of autism). The studies whose data we used were careful to match the age and sex of their control groups to their case population (GSE18123, GSE28475, GSE38322, GSE6575 and GSE37772). In addition, studies with differences of age and/or gender between cases and control groups independently performed statistical analyses to confirm that neither age nor sex impacted the pattern of differential gene expression exhibited between control and case individuals (GSE28521, GSE25507); GSE7329 and GSE39447 studies examined only males, although matched for age. More information regarding number of subjects, age, gender, ethnicity and relationship among the individuals in each original study can be found in Supplementary Table S1b.

### Cluster analysis

We then converted the transcriptional probe lists into a matrix of binary gene presence/absence with respect to each experimental case. We analyzed the matrix using the Jaccard coefficient in MATLAB (www.mathworks.com) to construct a gene-based dendrogram for all 27 biosets with the aim of determining the relatedness among them. This statistic measures the similarity and diversity among sample sets and is defined by the size of the intersection divided by the size of the union of sample sets. We elected to use this distance coefficient as it was originally developed for pattern discovery with binary matrices and does not treat the shared absence of a characteristic as evidence for relatedness, a valuable characteristic in the context of identifying similarity across gene expression experiments.

### Cluster bootstrap validation

We utilized an integrated function of the ‘fpc' package^28^ in R (http://www.R-project.org/), clusterboot(), for the assessment of clusterwise stability and validity of the clusters within the gene expression tree. We used a non-parametric bootstrapping procedure (*B*=1000 runs) to resample from the original data with replacement to construct bootstrap matrices and clusters, and iteratively used the Jaccard coefficient to compute the structural similarity of the resampled trees with the tree derived from the original data. We treated the mean of the Jaccard coefficients computed per permutation as the overall similarity between the original and permuted data, therefore an index of a cluster's stability. For each permutation, we set the paremeter *k*, the number of subsets, to 14 to match the total number of clusters obtained in the observed gene expression tree. Clusters supported by a Jaccard coefficient above 0.6 were treated as robust and stable, with values closer to 1.0 having the highest stability. Values ⩽0.5 were not stable and not considered in our analysis.

A more detailed explanation of the approach and justification for the cutoffs used here can be found in the study by Hennig.^29^ These cluster stability analyses were complemented with a classical multidimensional scaling approach that projects our dissimilarity data onto its first two principal dimensions, generated by the ‘showplots' argument of Clusterboot() function. This resulted in a series of clusters including a collection of 66 genes that form the root of a statistically significant cluster involving both blood and brain experiments, and that we hereafter refer to as Root 66.

### Gene annotation

To assess the biological context and potential significance of the Root 66 cluster, we used a manually curated database of genes linked to ASD, SFARI Gene^30^ and a systems medicine tool, Autworks,^31^ to extract information of autism candidate genes and candidate genes of related neuropsychiatric diseases together with an updated set of predicted gene interactions.

We also conducted a manual search for lists of variants that have been associated with autism candidate genes to increase coverage and reliability of the variant data used for validation of the biological significance of the Root 66 gene set.^32, 33, 34, 35, 36^

### Functional and gene-network analyses

We performed biological pathway analysis using Ingenuity Pathway Analysis (IPA) software (Ingenuity Systems, www.ingenuity.com) to explore gene connectivity and related biological functions both within and across the disorders. IPA functional analysis associates biological functions and diseases with experimental results, including differentially expressed genes selected from microarray experiments. It leverages the biological interactions deposited in the manually curated Ingenuity Pathways Knowledge Base and organizes this information to provide statistical support for gene-to-gene associations. We imported corresponding Root 66 gene symbols into the Ingenuity Pathways Knowledge Base and generated networks using an edge rank score (*P*-score=−log10(*P*-value)) that indicated the likelihood of the genes co-occurring or interacting by random chance. A score >3 (*P*<0.001) suggested with more than 99.9% confidence that an edge between two genes was non-random. Finally, we carried out a ‘disease and function' analysis to test whether the Root 66 genes had overrepresentation in specific human diseases and to explore the role(s) of the Root 66 genes in the context of statistically relevant biological processes pathways, and networks; IPA implemented a Fisher's exact test to generate *P*-values to determine whether a biological process was enriched with genes of interest.

### Comparison with other human disease gene expression

All RNA human expression disease vs normal experiments from Nextbio were manually curated for further analysis. In all, 1047 biosets were selected from 450 different experiments to perform pairwise intersections of the differentially expressed genes obtained in each experimental case–control comparison. We generated a matrix of the number of Root 66 genes present in each pairwise comparison using a custom Python script, and used RStudio^37^ to statistically explore the data sets and to build a distribution showing the frequency of Root 66 overlap for each bioset intersection.

### Comparison with patterns of gene expression in normal tissue

We conducted a manual literature search to collect and annotate major gene expression studies^38, 39, 40, 41, 42^ of normal human tissues with the aim of investigating evidence of genes in the Root 66 gene set being involved in normal blood and/or brain transcriptional patterns. In addition, we assembled a manually curated list of 408 housekeeping genes from Reactome and KEGG pathway annotations^43^ to test whether Root 66 genes have previously been found to be widely expressed across normal tissue and involved in housekeeping roles.

## Results

### Comparative analysis of gene expression studies

**Figure 1:**
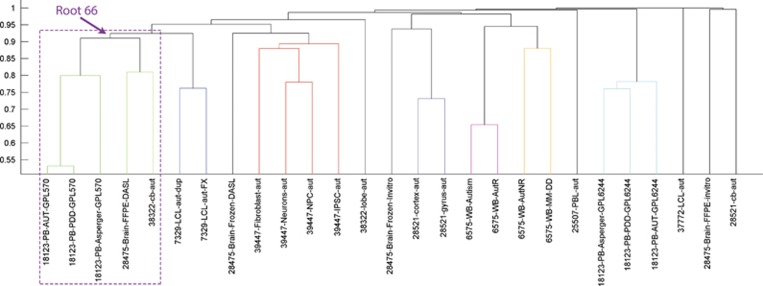
Gene-based clustering of the 27 biosets (see Supplementary Table S1). The majority clustered together by experiment first and tissue type second, with the exception of the Root 66 subgroup (highlighted in purple).

**Figure 2:**
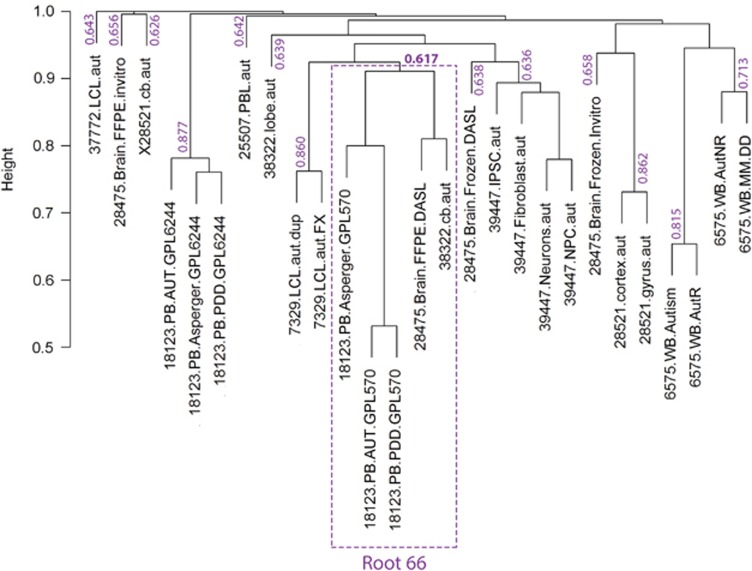
Jaccard clustering of the 27 biosets generated by bootstrap with replacement (*k*=14). The Root 66 subgroup, highlighted in purple, presented a stability index value of 0.617, suggesting that it was a non-random group of probable biological significance.

We generated lists of differentially expressed genes from ASD gene expression data analyses of 27 case–control biosets spanning 9 independent experiments: GSE37772, GSE25507, GSE7329, GSE28475, GSE38322, GSE6575, GSE18123, GSE28521 and GSE39447,^7, 12, 13, 14, 15, 16, 17, 18, 19^ available via Nextbio.^11^ Supplementary Table S1a provides a description of all biosets and Supplementary Table S1b contains information regarding number of subjects used in the experiments along with their age, gender and ethnicity. To determine relatedness across biosets, we converted the lists into a binary matrix of gene presence/absence and performed distance-based clustering with pairwise similarity measured via the Jaccard coefficient. As expected, the majority of the 27 biosets clustered together by experiment first and tissue type second (Figure 1, also Supplementary Figure S1), suggesting that the likely cause of the significant clustering of these biosets was variation related to the experimental design and nature of the samples. However, one subgroup of the tree deviated from this expectation and included biosets from three different experiments (GSE18123, GSE38322 and GSE28475) involving both brain and blood tissue (Figure 1). This subgroup was rooted by a collection of 66 genes (see Table 1 and Supplementary Table S2) and was statistically supported by bootstrap resampling (see Figure 2, also Supplementary Figure S2 and Supplementary Table S3). It is important to note that this Root 66 cluster was the only one that achieved statistical significance that integrated 3 different experiments from different tissues (brain and blood) across 3 independent expression platforms (GPL570 from Affymetrix (Santa Clara, CA, USA) and GPL6883 and GPL10558 from Illumina (Hayward, CA, USA)) and a diversity of subjects included (ASD and control males and females with different ages). This suggested that the source of the clustering was not co-variables but instead the regulatory patterns represented by these 66 genes.

To evaluate the hypothesis that this Root 66 could represent a common signature in blood and brain indicative of autism, we examined the genetic network formed by these genes, their implication in other autism-related neurological conditions and their mutational burden as reported by recently published exome-sequencing studies.^32, 33, 34, 35, 36^

### Links to autism

Four genes in our candidate list for autism (MAP1LC3B, PDE4B, TCF4 and UPF2) have already been associated with ASD. In addition, 32 interact directly with genes that have been associated with autistic disorder. An additional 19 are directly involved in or have links with genes associated with related neurological conditions, including schizophrenia, epilepsy, intellectual disability, seizures, attention deficit and disrupted behavior disorders, Angelman syndrome, bipolar disorder, mental retardation, developmental disabilities, sleep disorders and Alzheimer's disease, among others (Table 1). Supplementary Table S2 details the Root 66 genes including links to neurological disorders and interaction with known autism genes. Triangulating with recently published exome-sequencing studies, nine of the Root 66 genes have had variants reported as *de novo* or elevated risk for autism,^32, 33, 34, 35, 36^ and Table 2.

### Pathway enrichment

Using IPA, we determined that the Root 66 gene set was enriched, with IPA edge rank scores >3, in a total of 3 biological networks related to neurological function. These are detailed in the three sections below:

#### Neuroendocrine and normal development network (IPA score=27, *P*≪0.01)

The first network (Supplementary Figure S3) included 15 Root 66 genes, 2 of them already associated with autism (MAP1LC3B and PDE4B), that interact in neurological processes involved in normal brain growth and function, such as formation of astrocytes, proliferation of cortical neurons, sorting of axons and myelination. Dysregulation of Root 66 genes may affect relevant brain processes such as learning and memory, as they were found to be linked to relevant nodes in the network (PI3K and NFKB complexes) that are implicated in postsynaptic density and glutamatergic synapses, and, therefore, in synaptic plasticity. This network also showed an interesting connection between genes associated with ASD and other related neurological disorders, and endocrine hormones of the hypothalamic–pituitary–gonadal axis, such as follicle-stimulating hormone, luteinizing hormone and gonadotropin-releasing hormone.

#### Neurodegeneration network (IPA score=24, *P*≪0.01)

A second network (Supplementary Figure S4) included 14 Root 66 genes (UPF2 already linked to autism) that have been shown to interact with molecules involved in mechanisms responsible for nervous system inflammation, loss of neurological function and abnormal morphology of the brain. Several genes central in the network, such as APP, PTGS2, ERG and YWHAG were found to be linked to Root 66 genes, and have been implicated in processes such as amyloid plaque formation, astrocytosis and gliosis, cognitive degeneration and neurological dysfunction typical of neurological diseases including Parkinson's, Alzheimer's and Schizophrenia.

#### Neurodegeneration and tumor network (IPA score=22, *P*≪0.01)

Thirteen Root 66 genes were found to be interacting in this network (Supplementary Figure S5). Three genes involved in cancer appeared to be the most connected nodes: TP53, PTEN and AGTR1. The network was enriched in biological processes such as neurodegeneration, abnormal morphology and damage of the nervous system, likely caused by tumorigenesis.

**Figure 3:**
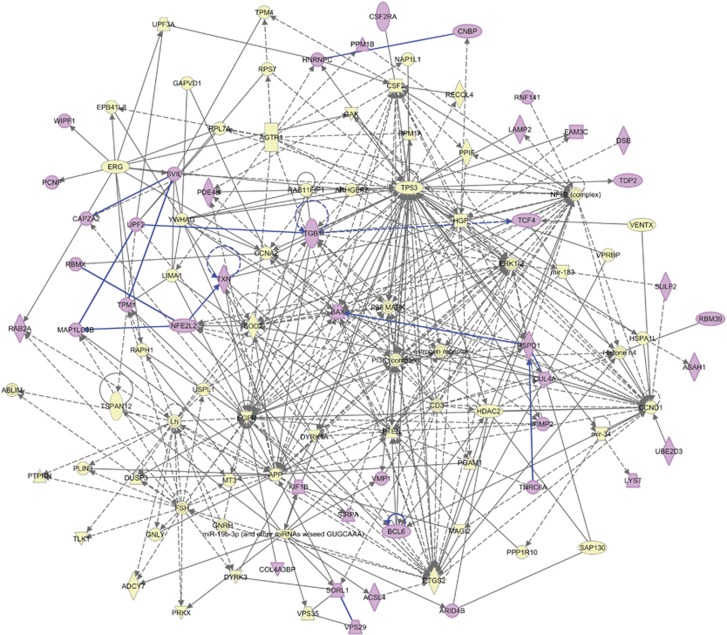
Biological network formed by the Root 66 gene set. Forty-two Root 66 genes (highlighted in purple) are tightly connected and interact in biological processes related to neurological conditions indicated in synaptic transmission, neurodegeneration, abnormal brain morphology, and learning and memory (Table 3). Interactions with any of the Root 66 genes are highlighted in blue.

While the above 3 networks showed potentially independent roles of smaller sets of genes contained with the Root 66, we also queried the global connectivity among member genes of Root 66. To do so, we merged the three networks into a single global gene network (Figure 3). The merged network connected 42 of the Root 66 genes (Figure 3) and revealed direct links between these Root 66 genes and genes participating in pathways related to neurological processes, such as synaptic transmission, neurodegeneration, learning and memory, as described above in the independent networks and labeled in Supplementary Figures as provided by IPA. Several autism-related diseases and functions were statistically enriched in this global network; gene names and corresponding *P*-values can be found in Table 3. We found evidence to support the role of the Root 66 network in biological mechanisms that cause alteration of the nervous system, and, therefore, neurological disorders, and also involvement in morphologic and molecular alterations of normal brain development that may underlie the etiology of ASD. While this global network was statistically significant, more testing will be required to confirm its biological significance and potential role in autism.

### Root 66 expression in non-autism disease and normal tissue

We manually selected 1047 biosets from Nextbio^11^ containing genes differentially expressed between disease and normal tissue from 450 independent RNA expression experiments. These experiments contained a diversity of tissue types roughly comparable to the experiments used in our primary analysis. We then computed pairwise intersections between these biosets to assess the frequency of appearance of our Root 66 in each overlap. The distribution displayed in Supplementary Figure S6 details the number of Root 66 genes found in each pairwise bioset intersection. The overlap was negligible and not statistically significant. The average number of genes found in the intersection among these biosets was 6.15 compared with the 66 genes obtained in individuals with autism. In addition, we found five relevant studies^38, 39, 40, 41, 42^ where hierarchical clustering of normal tissues was made using different approaches; none of these showed a grouping between brain and blood samples. Instead, these two tissue types segregated separately into unconnected branches within the dendrogram. In addition, only 3 Root 66 genes (RBMX, SUPT4H1 and UBE2D3) intersected with our list of 408 experimentally confirmed housekeeping genes.^43^ These findings collectively lend support to the hypothesis that the genes in the Root 66 cluster play unique roles in autism.

## Discussion

In this study, we compared a large set of published and openly available gene expression experiments from different tissue types performed in individuals with autism, with the goal of testing the hypothesis that a signature of autism can be found in the blood that might be a molecular echo of autism-related regulatory impairment in the brain.

We discovered a statistically significant subgroup that deviated from expectation and included biosets from three independent experiments involving both brain and blood tissue. This subgroup was significantly statistically supported by a subset of 66 genes that we termed Root 66. Four of the Root 66 genes (MAP1LC3B, PDE4B, TCF4 and UPF2) have previously been associated with autism, and 56 either have been shown to interact directly with known autism candidates or have been implicated in other autism-related neurological disorders.

To better understand the biological significance of Root 66, we tested its member genes' enrichment in specific biological processes and whether these genes form an interconnected biological network. Our analysis revealed that a significant number of Root 66 genes were interacting in 3 relevant networks, each of them related to neurological disease in a different but potentially significant way. The first network was enriched in biological processes related to brain growth and development that may affect learning and memory, and also showed some dysregulation in neuroendocrine activity, particularly an interaction between Root 66 genes and endocrine hormones of the hypothalamic—pituitary—gonadal axis. Although the main role of this axis is to control development, reproduction and aging, it is known that these hormones affect behavior, as they have been shown to alter brain structure and functioning including dsyregulation of the follicle-stimulating hormone, which has known roles in brain development and neuronal differentiation.^44^ Moreover, the activity of gonadotropin-releasing hormone neurons, and thus, the regulation of gonadotropin release in blood, is stimulated or inhibited by oxytocin, a neurohypophysial hormone that also acts as a neurotransmitter in the brain. Several studies^45, 46, 47, 48, 49^ have revealed that oxytocin is implicated in social behavior, recognition and bonding, as well as the establishment of trust among people. Furthermore, there is evidence that alterations in the neuromodulatory role of oxytocin are linked to a variety of mental disorders, including autism.^50, 51, 52^ Finally, polymorphisms in the gene that codes for the oxytocin receptor have been associated with ASD risk.^53^

The second network showed Root 66 gene enrichment in nervous system inflammation, loss of neurological function and abnormal morphology of brain, supporting roles in neurodegeneration. There is evidence^54, 55^ of neural cell loss and activation of microglia and astrocytes in ASD, as well as high levels of APP.^56, 57, 58^ These studies suggested that neurodegeneration may have a role in autism, as it could explain the loss of previously acquired skills and abilities indicative of regressive forms of autism. Alterations in common neurological mechanisms, such as disruption during synaptogenesis may relate to ASD and other disorders of the brain, including schizophrenia, epilepsy, Alzheimer's disease and Parkinson's disease. For example, work has shown evidence for impaired neural synchrony and neurotransmission systems as pathophysiological processes involved in onset and/or maintenance of these neurological conditions.^59, 60, 61, 62, 63, 64^

Finally, the third network was enriched in neurodegeneration, abnormal morphology and damage of the nervous system and included the known cancer genes TP53, PTEN and AGTR1 as the top most connected nodes. Mutations in tumor suppressor gene PTEN, known to be associated with thyroid, breast and colon cancers, have been found in subgroups of children with autism who also have comorbid conditions of macrocephaly and/or epilepsy.^65, 66^ In addition, it has been suggested that PTEN mutations can have downstream impacts on other autism gene candidates, perhaps playing a role in the autism phenotype.^67^ Work has shown that when defective PTEN interacts with TP53 a decrease in the energy production of neurons occurs, leading to stress that induces mitochondrial DNA changes and abnormal levels of energy production in brain regions that are crucial for social behavior and cognition.^68^

Merging of these networks revealed that Root 66 genes formed a tightly connected network enriched in neurological processes such as synaptic transmission, neurodegeneration, abnormal brain morphology, learning and memory, supporting its potential role in neurological impairment, and in particular, autism. We next ran several tests to determine whether Root 66 represented a unique signature in autism unlikely to occur by chance or to represent a more ubiquitous signature of neurological impairment. We explored the list of differentially expressed genes in 450 disease vs normal RNA expression experiments for overlap with Root 66. The mean overlap of 6 genes strongly supported the hypothesis that the brain–blood cluster formed by Root 66 was unlikely to be a chance event and may represent a pattern unique to autism.

To further test whether the Root 66 brain–blood cluster observed in our study could form by chance, we explored the results from 5 studies^38, 39, 40, 41, 42^ of normal brain and blood gene expression. These studies showed that although there is overlap in the genes expressed in normal brain and blood, the different tissue transcriptomes clustered independently from one another in all cases, unlike what we observed in the present analysis. We also tested whether the genes in Root 66 played more generic roles as housekeeping genes.^69^ Only 3 of its member genes overlapped with an experimentally confirmed list of 408 housekeeping genes manually curated from Reactome and KEGG pathway databases.^43^ Both results lend additional support to the hypothesis that the Root 66 cluster is non-random and likely plays a role unique to autism.

## Conclusion

Gene expression studies published to date have had a relatively limited impact on our understanding of autism's molecular pathology. Here we show by integrating and analyzing several published gene expression experiments a statistically significant signal between blood and brain rooted by 66 genes. The Root 66 gene set appeared to be non-random and of potential etiologic relevance to autism, as most of its members have known association with neurological processes crucial for normal brain development and function and confirmed roles in neurological disease. While further independent replication and experimental validation will be needed to confirm our preliminary findings, the current results suggest that there is a detectable signature in the blood of individuals with autism that echoes what might be an important signature of dysregulation in the brain.

## Figures and Tables

**Table 1:** Root 66 genes (1) with known links to autism, (2) known to be interacting with high priority autism candidates or (3) associated with other autism-related neurological disorders

*Root 66 genes already associated to autism*	*Root 66 interacting with ASD candidate genes*	*Root 66 directly involved or interacting with genes linked to related neurological conditions*^a^
*MAP1LC3B, PDE4B, TCF4, UPF2*	*ACSL4, ASAH1, BAX, BCL6, CAPZA2, CNOT4, COL4A3BP, CSF2RA, CUL4A, EIF4E3, GMPR2, HNRNPC, HSPD1, ITGB1, LAMP2, LYST, NFE2L2, PCNP, PFDN5, PPM1B, RBM39, RBMX, RHEB, SEPT2, SIRPA, SVIL, TDP2, TIMP2, TPM1, TXN, UBE2D3, ZNF644*	*AGTPBP1, ARID4B, CPD, DSE, KIF1B, LAMTOR3, OCIAD1, PHF20L1, PNKD, RAB24, RAB2A, RBM25, SLC44A2, SORL1, SUPT4H1, TNRC6A, VMP1, WIPF1, WLS*

Abbreviation: ASD, autism spectrum disorder.

a Schizophrenia, epilepsy, intellectual disability, seizures, attention deficit hyperactivity disorder, Angelman syndrome, bipolar disorder and Alzheimer's disease

**Table 2:** Root 66 genes with SNVs or *de novo* ASD risk-contributing mutations in ASD probands from several recently published exome-sequencing efforts

*Study*	*Source*	*Gene/type mutation*
Iossifov *et al.*^32^	Complete list of SNVs detected on 343 SSC families	*PDE4B*: autism F, synonymous *RAB2A*: autism M, synonymous *BCL6*: autism M, synonymous
Neale *et al.*^33^	Validated *de novo* events and mutations	*SVIL*: autism, nonsense
ASD genes	*ACSL4*, *LAMP2*	
O'Roak *et al.*^34^	Top *de novo* ASD risk-contributing mutations	*CNOT4*
*De novo* mutation sites	*CNOT4*: missense *TCF4*: synonymous *ZNF644*: missense, severe	
O'Roak *et al.*^35^	ASD candidate loci targeted by MIPs- inherited truncation/splice events identified in ASD probands	*CNOT4*
Sanders *et al.*^36^	Loss-of-function mutations in probands	*RAB2A*

Abbreviations: ASD, autism spectrum disorder; MIP, molecular inversion probe; SNV, single nucleotide variant; SSC, Simons Simplex Collection.

**Table 3:** Significant diseases and functions enriched in the Root 66 biological network (Figure 3)

*Diseases and function*	P*-value*	*Molecules*
Alzheimer's disease	5.66E−10	APP, **BAX**, DYRK1A, estrogen receptor, **FAM3C**, GAK, GAPVD1, **HSPD1**, miR-19b-3p, mir-34, **NFE2L2**, NFkB (complex), PTEN, PTGS2, SOD2, **SORL1**, TP53, **TXN**, VPS35
Morphology of nervous system	7.53E−10	AGTR1, APP, **BAX**, CCND1, DYRK1A, EGFR, EPB41L3, GAK, HDAC2, **ITGB1**, **KIF1B**, MAGI2, mir-34, **NFE2L2**, PTEN, SOD2, **SORL1**, **SULF2**, TP53
Size of brain	2.58E−08	**BAX**, DYRK1A, EGFR, **KIF1B**, PTEN, SOD2, TP53
Abnormal morphology of nervous system	3.62E−08	AGTR1, APP, **BAX**, CCND1, DYRK1A, EGFR, EPB41L3, HDAC2, **KIF1B**, MAGI2, **NFE2L2**, PTEN, SOD2, **SORL1**, **SULF2**, TP53
Morphology of brain	4.45E−08	AGTR1, APP, **BAX**, CCND1, DYRK1A, EGFR, GAK, HDAC2, **KIF1B**, PTEN, SOD2, **SULF2**, TP53
Differentiation of neurons	9.09E−08	**ACSL4**, APP, **ASAH1**, **BCL6**, EGFR, HDAC2, HGF, **ITGB1**, PI3K (complex), **TCF4**, **TIMP2**, TP53, YWHAG
Abnormal morphology of brain	1.82E−07	AGTR1, APP, **BAX**, CCND1, DYRK1A, EGFR, HDAC2, **KIF1B**, PTEN, SOD2, **SULF2**, TP53
Morphogenesis of neurites	2.00E−07	APP, DYRK1A, EGFR, EPB41L3, HGF, **ITGB1**, MAGI2, mir-34, PTEN, RAPH1, **SULF2**, TP53
Neuritogenesis	3.11E−07	APP, DYRK1A, EGFR, EPB41L3, ERK1/2, HDAC2, HGF, **ITGB1**, MAGI2, mir-34, PI3K (complex), PTEN, RAPH1, **SIRPA, SULF2**, TP53
Development of neurons	3.23E−07	ABLIM, APP, DYRK1A, EGFR, EPB41L3, ERK1/2, HDAC2, HGF, **ITGB1**, MAGI2, mir-34, PI3K (complex), PTEN, RAPH1, **SIRPA**, SOD2, **SULF2**, TP53, YWHAG
Branching of neurites	3.36E−07	APP, DYRK1A, HGF, **ITGB1**, MAGI2, mir-34, PTEN, RAPH1, **SULF2**, TP53
Shape change of neurons	6.27E−07	APP, DYRK1A, HGF, **ITGB1**, MAGI2, mir-34, PI3K (complex), PTEN, RAPH1, **SULF2**, TP53
Morphology of neurons	1.46E−06	APP, **BAX**, DYRK1A, EGFR, EPB41L3, HDAC2, MAGI2, mir-34, **NFE2L2**, **SORL1**, **SULF2**
Abnormal morphology of neurons	1.84E−06	APP, **BAX**, DYRK1A, EGFR, EPB41L3, HDAC2, MAGI2, **NFE2L2**, **SORL1**, **SULF2**
Proliferation of neural precursor cells	2.41E−06	APP, DYRK1A, HGF, mir-34, PTEN
Size of dendritic trees	2.47E−06	**BAX**, HGF, **ITGB1**

Abbreviation: IPA, Ingenuity Pathway Analysis.

Root 66 genes highlighted in bold. *P*-values were generated by IPA using Fisher's exact test.
