# Supplementary material for: A common molecular signature in ASD gene expression: following Root 66 to autism
Source: Transl Psychiatry. 2016 Jan 5;6(1):e705–. doi: 10.1038/tp.2015.112 (PMC5068868; doi:10.1038/tp.2015.112)
Supplement: Supplementary Information [file tp2015112x1.doc]

| Bioset Name | **Experiment** | **Platform** | **Nextbio Bioset Name** | **# Nextbio Replicates** | **# Diferentially Expressed Genes** | **Sample Type** | **Primary Reference** |
| --- | --- | --- | --- | --- | --- | --- | --- |
| 18123 PB Aut GPL570 | GSE18123 | GPL570 | Autism patients *vs* healthy controls | 42 *vs* 55 | 3061 | Peripheral Blood | Sek Wom Kong *et al*., 2012. PMID: 23227143 |
| 18123 PB PDD GPL570 |  |  | PDD-NOS patients *vs* healthy controls | 38 *vs* 55 | 2020 |  |  |
| 18123 PB Asperger GPL570 |  |  | Asperger Patients *vs* healthy controls | 10 *vs* 55 | 1733 |  |  |
| 18123 PB Aut GPL6244 |  | GPL6244 | Autism patients *vs* healthy controls | 41 vs 82 | 257 |  |  |
| 18123 PB PDD GPL6244 |  |  | PDD-NOS patients *vs* healthy controls | 48 *vs* 82 | 644 |  |  |
| 18123 PB Asperger GPL6244 |  |  | Asperger patients *vs* healthy controls | 15 *vs* 82 | 557 |  |  |
| 7329 LCL Aut fx | GSE7329 | GPL1708 | Male autistic patients with Fragile X mutation *vs* healthy controls | 8 vs 27 | 2221 | Lymphoblast Cell Lines | Yuhei Nishimura *et al*., 2007. PMID: 17519220 |
| 7329 LCL Aut dup |  |  | Male autistic patients with 15q11-q13 duplication *vs* healthy controls | 7 vs 27 | 1639 |  |  |
| 28475 Brain FFPE DASL | GSE28475 | GPL6883 | Brain samples frozen and formalin-fixed cDNA-mediated annealing, selection and ligation assay autistic patients *vs* healthy controls | 4 *vs* 16 | 4654 | Brain | Maggie L. Chow *et al*., 2012. PMID: 22375143 |
| 28475 Brain Frozen DASL |  |  | Brain samples frozen cDNA-mediated annealing, selection and ligation autistic patients *vs* healthy controls | 38 *vs* 36 | 2431 |  |  |
| 28475 Brain Frozen In Vitro |  |  | Brain samples frozen in vitro assay autistic patients *vs* healthy controls | 9 *vs* 4 | 1151 |  |  |
| 28475 Brain FFPE In Vitro |  |  | Brain samples frozen and formalin-fixed in vitro assay autistic patients *vs* healthy controls | 1 *vs* 5 | 132 |  |  |
| 38322 cb autism | GSE38322 | GPL10558 | Cerebellum autistic patients *vs* healthy controls | 14 vs 12 | 3642 | Cerebellum | M. R. Ginsberg *et a*l., 2012. PMID: 22984548 |
| 38322 lobe autism |  |  | Occipital lobe autistic patients *vs* healthy controls | 4 *vs* 6 | 1041 | Brain |  |
| 39447 fibroblast aut | GSE39447 | GPL13635 | Fibroblasts from autistic patients with BCKDK mutation *vs* healthy controls | 5 *vs* 2 | 3018 | Fibroblasts | Gaia Novarino *et al*., 2012. PMID:22956686 |
| 39447 Neurons aut |  |  | Neurons iPSC derived from autistic patients with BCKDK mutation *vs* healthy controls | 8 *vs* 4 | 5565 | Neurons iPSC derived |  |
| 39447 NPC aut |  |  | NPC iPSC derived from autistic patients with BCKDK mutation *vs* healthy controls | 6 *vs* 2 | 9040 | Neural Progenitor Cells iPSC derived |  |
| 39447 iPSC aut |  |  | iPSCs derived from fibroblasts autistic patients with BCKDK mutation *vs* healthy controls | 8 *vs* 4 | 2536 | Induced pluripotent stem cells derived from fibroblasts |  |
| 28521 cortex aut | GSE28521 | GPL6883 | Prefrontal cortex of ASD patients *vs* healthy donor | 16 *vs* 16 | 850 | Brain | Irina Voineagu *et al*., 2013.  PMID: 21614001 |
| 28521 gyrus aut |  |  | Superior temporal gyrus ASD patients *vs* healthy donor | 13 *vs* 13 | 722 |  |  |
| 28521 cb aut |  |  | Cerebellar vermis of ASD patients *vs* healthy donor | 10 *vs* 11 | 116 | Cerebellum |  |
| 6575 WB Aut | GSE6575 | GPL570 | Autistic patients *vs* normal controls | 35 *vs* 12 | 335 | Whole Blood | Jeffrey P. Gregg *et al*., 2008. PMID:18006270 |
| 6575 WB AutNR |  |  | Autistic patients with no regression *vs* normal controls | 17 *vs* 12 | 1120 |  |  |
| 6575 WB AutR |  |  | Autism patients with regression *vs* normal controls | 17 *vs* 12 | 404 |  |  |
| 6575 WB MRDD |  |  | Patients with mental retardation or developmental delay (MRDD) *vs* normal controls | 9 *vs* 12 | 602 |  |  |
| 25507 PBL aut | GSE25507 | GPL570 | Autistic children *vs* normal controls | 82 *vs* 64 | 165 | Peripheral Blood Lymphocytes | M.D. Alter *et al*., 2011  PMID: 21379579 |
| 37772 LCL aut | GSE37772 | GPL6883 | Autistic patients *vs* control family members | 195 *vs* 195 | 7 | Lymphoblast Cell Lines | Rui Luo *et al*., 2012  PMID: 22726847 |

**Supplementary Table S1a**. Description of the 27 biosets.

| **Experiment** | **Platform** | **# Individual Samples Original Datasets** | | Gender | | | **Mean age (years)** | | | | **Race/ethnicity**  **(# individuals)** | **Bioset Name** | **Nextbio Bioset Name** | **# Nextbio Replicates** |
| --- | --- | --- | --- | --- | --- | --- | --- | --- | --- | --- | --- | --- | --- | --- |
|  |  | **Case** | **Control** | Case | Control | | **Case** | | **Control** | |  |  |  |  |
| GSE18123 | GPL570 | 31 autism | 33 | Male | | | 8 | 9 | | | Asian (2) Black (6) Caucasian (72) Mixed (4) Other(4) Unknown (11) | 18123 PB Aut GPL570 | Autism patients *vs* healthy controls | 42 *vs* 55 |
|  |  | 26 PDD-NOS |  |  |  |  |  |  |  |  |  | 18123 PB PDD GPL570 | PDD-NOS patients *vs* healthy controls | 38 *vs* 55 |
|  |  | 9 Asperger |  |  |  |  |  |  |  |  |  | 18123 PB Asperger GPL570 | Asperger Patients *vs* healthy controls | 10 *vs* 55 |
|  | GPL6244 | 41 autism | 82 | 80 male  24 fem | | 48 male  34 fem | 8.4 | 8.1 | | | Asian (5) Black (8) Caucasian (129) Mixed (11) Other(21) Unknown (12) | 18123 PB Aut GPL6244 | Autism patients *vs* healthy controls | 41 vs 82 |
|  |  | 48 PDD-NOS |  |  |  |  |  |  |  |  |  | 18123 PB PDD GPL6244 | PDD-NOS patients *vs* healthy controls | 48 *vs* 82 |
|  |  | 15 Asperger |  |  |  |  |  |  |  |  |  | 18123 PB Asperger GPL6244 | Asperger patients *vs* healthy controls | 15 *vs* 82 |
| GSE7329 | GPL1708 | 8 autism FMR1-FM | 15 | Male | | | - | | | | Asian (2) Caucasian(18) Unknown (10) | 7329 LCL Aut fx | Male autistic patients with Fragile X mutation *vs* healthy controls | 8 vs 27 |
|  |  | 7 autism dup(15q) |  |  |  |  |  |  |  |  |  | 7329 LCL Aut dup | Male autistic patients with 15q11-q13 duplication *vs* healthy controls | 7 vs 27 |
| GSE28475 | GPL6883 | 28 autism | 29 | 22 male  6 fem | | 22 male  7 fem | 19.9 | 19.1 | | | - | 28475 Brain FFPE DASL | Brain samples frozen and formalin-fixed cDNA-mediated annealing, selection and ligation assay autistic patients *vs* healthy controls | 4 *vs* 16 |
|  |  |  |  |  |  |  |  |  |  |  |  | 28475 Brain Frozen DASL | Brain samples frozen cDNA-mediated annealing, selection and ligation autistic patients *vs* healthy controls | 38 *vs* 36 |
|  |  |  |  |  |  |  |  |  |  |  |  | 28475 Brain Frozen In Vitro | Brain samples frozen in vitro assay autistic patients *vs* healthy controls | 9 *vs* 4 |
|  |  |  |  |  |  |  |  |  |  |  |  | 28475 Brain FFPE In Vitro | Brain samples frozen and formalin-fixed in vitro assay autistic patients *vs* healthy controls | 1 *vs* 5 |
| GSE38322 | GPL10558 | 9 autism | 9 | Male | | | 22 | 22 | | | - | 38322 cb autism | Cerebellum autistic patients *vs* healthy controls | 14 vs 12 |
|  |  |  |  |  |  |  |  |  |  |  |  | 38322 lobe autism | Occipital lobe autistic patients *vs* healthy controls | 4 *vs* 6 |
| GSE39447 | GPL13635 | 3 autism | 2 | Male | | | 7.6 | | | - | Mediterranean  (Egyptian,  Turkish, Libyan)  Consanguineous families | 39447 fibroblast aut | Fibroblasts from autistic patients with BCKDK mutation *vs* healthy controls | 5 *vs* 2 |
|  |  |  |  |  |  |  |  |  |  |  |  | 39447 Neurons aut | Neurons iPSC derived from autistic patients with BCKDK mutation *vs* healthy controls | 8 *vs* 4 |
|  |  |  |  |  |  |  |  |  |  |  |  | 39447 NPC aut | NPC iPSC derived from autistic patients with BCKDK mutation *vs* healthy controls | 6 *vs* 2 |
|  |  |  |  |  |  |  |  |  |  |  |  | 39447 iPSC aut | iPSCs derived from fibroblasts autistic patients with BCKDK mutation *vs* healthy controls | 8 *vs* 4 |
| GSE28521 | GPL6883 | 19 autism | 17 | 14 male  5 fem | | 16 male  1 fem | 24 | 34.6 | | | Asian (2) Caucasian (34) | 28521 cortex aut | Prefrontal cortex of ASD patients *vs* healthy donor | 16 *vs* 16 |
|  |  |  |  |  |  |  |  |  |  |  |  | 28521 gyrus aut | Superior temporal gyrus ASD patients *vs* healthy donor | 13 *vs* 13 |
|  |  |  |  |  |  |  |  |  |  |  |  | 28521 cb aut | Cerebellar vermis of ASD patients *vs* healthy donor | 10 *vs* 11 |
| GSE6575 | GPL570 | 35 autism (R+NR) | 12 controls | 30m 5f | | 9 male  3 fem | 3.5 | 3.5 | | | Mainly Caucasian | 6575 WB Aut | Autistic patients *vs* normal controls | 35 *vs* 12 |
|  |  | 17 aut NR |  | 15m 2f | |  |  |  |  |  |  | 6575 WB AutNR | Autistic patients with no regression *vs* normal controls | 17 *vs* 12 |
|  |  | 18 aut R |  | 15m 3f | |  |  |  |  |  |  | 6575 WB AutR | Autism patients with regression *vs* normal controls | 17 *vs* 12 |
|  |  | 9 MRDD |  | 6m 3f | |  |  |  |  |  |  | 6575 WB MRDD | Patients with mental retardation or developmental delay (MRDD) *vs* normal controls | 9 *vs* 12 |
| GSE25507 | GPL570 | 82 autism | 64 | Male | | | 5.5 | 7.9 | | | Caucasian | 25507 PBL aut | Autistic children *vs* normal controls | 82 *vs* 64 |
| GSE37772 | GPL6883 | 233 autism | 206 | 199m  34 fem | | 107m  99 fem | 9.63 | 10.50 | | | Mainly Caucasian (3% African American) | 37772 LCL aut | Autistic patients *vs* control family members (Nextbio just included for the analysis 1^st^ stage individuals: 191 autism and 195 controls) | 195 *vs* 195 |

**Supplementary Table S1b**. Characteristics of subjects included in the studies.

| R**oot66 Gene Symbol** | Description | **Interesting Disease Networks** | **Interaction with Autism genes** | **Interactions with genes related to Neurological Disorders** |
| --- | --- | --- | --- | --- |
| ***ACSL4*** | acyl-CoA synthetase long-chain family member 4 | Pervasive Child Development Disorders, Depressive Disorder, Intellectual Disability, Asperger Syndrome, X-linked Mental Retardation, Mental Disorders, Immune System Diseases, Nervous System Diseases, Neurologic Manifestations, Neurodegenerative Diseases, Mental Disorders Diagnosed in Childhood, Neurobehavioral Manifestations, Mood Disorders | ATRX, FMR1, SLC25A14, SLC9A6, UBE2A | CPT1B, UBC, UBQLN2, ACADM, LPL, LIPG, UVRAG, NKRF, CPT2, CEL, ACOX3, ATP6AP2, RPGR, PGK1, LAMP2, PHF6, ELOVL1, STAG2, ACOX1, RPS6KA3, AMMECR1, RP2, RAP2C, PNPLA3, PPT1 , FADS2 |
| ***AGTPBP1*** | ATP/GTP binding protein 1 |  |  | CYCS |
| ***AKIRIN1*** | akirin 1 |  |  |  |
| ***ARID4B*** | AT rich interactive domain 4B (RBP1-like) | Brain Diseases, Metabolic Brain Diseases, Central Nervous System Diseases, Nervous System Diseases, Neurologic Manifestations, Neurodegenerative Diseases, Inborn Metabolic Brain Diseases |  | ARID4A , HDAC1 , HDAC2, SIRT1 |
| ***ASAH1*** | N-acylsphingosine amidohydrolase (acid ceramidase) 1 | Schizophrenia, Mental Disorders, Brain Diseases, Metabolic Brain Diseases, CNS Diseases, Epilepsy, Nervous System Diseases, Neurodegenerative Diseases, Schizophrenia and Disorders with Psychotic Features, Inborn Metabolic Brain Diseases | UGCG | UGT8, GALC, DEGS1, SPHK1, SMPD3, SGMS1, KDSR, GBA , CERK, ASAH2, SMPD1 |
| ***BAX*** | BCL2-associated X protein | Brain Death, Seizures, Autoimmune Diseases, Mental Disorders, Brain Diseases, CNS Diseases, CNS Infections, Cerebellar Diseases, Cerebellar Ataxia, Colonic Diseases, Epilepsy, Gastrointestinal Diseases, Immune System Diseases, Intestinal diseases, Nervous System Diseases, Neurologic Manifestations, Inflammatory Bowel Diseases, Neurodegenerative Diseases, Neurobehavioral Manifestations | CREBBP, TOMM20 | YWHAQ, HDAC6, BAK1, BCL2L2, UHRF2, PMAIP1, MOAP1, MAX, XRCC6, UVRAG, BCL2A1, TP53, BCL2, PRKCE, MYC, BID, IRF3, UBC, PIN1, ERN1, PARK7, SFN, APAF1, MCL1, RELA, EP300, BCL2L1, GSK3B, IL3RA, HSPD1, BBC3, JMY, BAD, CSF2RB, SP1, SIRT1, SLC25A4, VDAC1,TOMM40, SNCA |
| ***BCL6*** | B-cell CLL/lymphoma 6 | Down Syndrome, Mental Disorders, Brain Diseases, CNS Diseases, Gastrointestinal Diseases, Immune System Diseases, Intellectual Disability, Nervous system Diseases, Mental Disorders Diagnosed in Childhood, Neurobehavioral Manifestations, | CREBBP, FCER2, MBD3 | MAPK1, TP53, KMT2D, ERCC5, JUN, RUNX1T1, CDK8, CDK19, BCOR, EHMT1, TCF7, SMARCA4,EIF2AK3, FOXM1, ARNT2, IRF4, SKP1, ELF1, HBEGF, FOXO4, EPHB6, RAC2, HNF1A, EBF1, ARID1A, FOXO3, BCL11A , NCOR2, PPARD, UBC, PIN1, SMO, HDAC5, ATF7, CHD3, SPI1, EP300, HDAC7, NKRF, HDAC1, HDAC4, YY1, HDAC2, SP1, HDAC9, PPARGC1A ,MAPK8IP3, ZBTB16, EIF4ENIF1, WNK4, SAE1,CACNA1A, PML,ZGLP1, NCOR1 PCDH9 |
| ***CAPZA2*** | capping protein (actin filament) muscle Z-line, alpha 2 | Nutrition Disorders, Nutritional and Metabolic Diseases | APC, APP | MORF4L1, LRRC16A , CAPG, RAB1A, ADD1, MMADHC, S100B, PRKAR1A, GMFB |
| ***CNBP*** | CCHC-type zinc finger, nucleic acid binding protein | Mental Disorders, Brain Diseases, Central Nervous System Diseases, Nervous System Diseases, Neurologic Manifestations, Neurodegenerative Diseases |  |  |
| ***CNOT4*** | CCR4-NOT transcription complex, subunit 4 |  | EIF4E, KDM5C | EIF4G1 |
| ***COL4A3BP*** | collagen, type IV, alpha 3 (Goodpasture antigen) binding protein | Autoimmune Diseases, Immune System Diseases, Endocrine System Diseases, Lupus Erythematosus, Systemic | CBS | CSNK1G2, RTN3, MARK2, COL4A3, PARP2, BCS1L |
| ***CPD*** | carboxypeptidase D | Mental Disorders, Nervous System Diseases, Neurodegenerative Diseases |  | UHRF2, TCF3, LCK |
| ***CSF2RA*** | colony stimulating factor 2 receptor, alpha, low-affinity (granulocyte-macrophage) | Schizophrenia, Mental Disorders, Brain Diseases, CNS Diseases, Gastrointestinal Diseases, Intestinal Diseases, Nervous System Diseases, Inflammatory Bowel Diseases, Schizophrenia and Disorders with Psychotic Features | HRAS, JAK2, PIK3CA, STAT3, YWHAZ | CBL, KIT, JAK3, NRAS, GRB2, CSF2RB, SOS1, PIK3R1, SOCS4, CBLB, PTPN6, IKBKB, SOCS2, SHC1, SOCS5, PTPN11, STAT1, SDC2, SOCS1, SOCS3, PRKACG, TYK2, JAK1, ITGB1, CSF2, RPSA, SYK, ITGA9 |
| ***CUL4A*** | cullin 4A | Immune System Diseases | TLE2, RFWD2 | TP53, NACC1, CDT1, DNMT3B, UBC, PTGS2, HSPA1A, MYC, PCNA, CRBN, EED, ERCC8, COPS3, CHEK1, GPS1, TOR1AIP2, KEAP1, NEDD8, AMBRA1, HOXA9, COPS5, DDB2, GLMN, KAT2A, HSP90AA1, BTRC, RBBP7, TRPC4AP, BANF1, COPS8, WDTC1, MDM2, CDKN1A, WDTC1, MDM2, CDKN1A, SKP2, RAG1, DDIT4, CTNNB1, NF2, CDKN1B, RNF7, COMMD1, TUBA1B, EMD, CUL4B |
| ***DSE*** | dermatan sulfate epimerase | Inborn Genetic Diseases |  | DCN,VCAN, BCAN, NCAN, BGN, CSPG5, CSPG4 |
| ***EIF4E3*** | eukaryotic translation initiation factor 4E family member 3 | Inborn Genetic Diseases, Congenital, Hereditary, and Neonatal Diseases and Abnormalities, Chromosome Disorders | EIF4E, FMR1, CYFIP1 | AGO2, ARIH1,ISG15, EIF4G1, EIF4G2 |
| ***FAM198B*** | family with sequence similarity 198, member B |  |  |  |
| ***FAM3C*** | family with sequence similarity 3, member C | Musculoskeletal Diseases |  |  |
| ***GMPR2*** | guanosine monophosphate reductase 2 |  | PDE1A | ATIC, PDE2A, PDE3A, PDE6B, PDE11A, PDE6C, PDE6A, PDE5A, ENTPD4, ADSS, APRT, PDE1B, PDE6H, PDE4A, NT5E, HPRT1, AMPD1, IMPDH1, NT5C3A, AMPD2, PDE9A, GUK1, PDE4D, PDE4B, PDE7A, PDE7B, ADSSL1, NT5C2, PDE4C, ENTPD1, PDE1C |
| ***HNRNPC*** | heterogeneous nuclear ribonucleoprotein C (C1/C2) | Prenatal Injuries, Immune System Diseases | TADA2A, TOP1, YWHAZ, UPF3B | EIF2AK2, MGMT, POLR2A,POLR2D, GRB2, UBC, CDKN2A, SRSF4, KRAS, CSNK1A1, ARHGAP4, PHF5A, FUS, HNRNPU, U2AF2, CPSF1, DKC1, YBX1, SH3KBP1, PABPN1, PDGFB, PTBP1, DHX38, SMC1A, SMC1A, RBM5, SMARCA4, CPEB1, SRSF9, FXR2, SRSF1, BRINP1, CD2BP2, BANF1, ALYREF, KHDRBS3, CHD4, PCBP2, KPNA3, POLR2G, TERT, PRKDC, SRSF2, UPF3A, SRSF5, PTBP2, SNRNP70, PRPF6, HNRNPD, POLR2F |
| ***HSPD1*** | heat shock 60kDa protein 1 (chaperonin) | Epilepsy, Intellectual Disability, Neurodegenerative diseases, Mood disorders, Mental Disorders, Sleep Disorders, Brain Diseases, CNS Diseases, Neurologic Manifestations, Neurobehavioral Manifestations, Mental Disorders Diagnosed in Childhood | APP, ATP5A1, DHFR, DNM1L, LRPPRC, TOMM20 | DLD, EDF1, DDX1, MSH2, MSH6, ATIC, MRPL3, TP53, LY96, CA2, FDXR, BAX, ABCE1, CASP3, GCSH, PPP2R2B, KAT5, UBC, TRRAP, MT-CO1, PGK1, HDAC1, TLR4, MYC, PCNA, GFM1, ITGA3, AHCY, TLR2, HSP90B1, HSPA5, FHIT, HNRNPA2B1, EIF3D, APEX1, STUB1, FBL, HSPA8, RARS, ETFA, CCT5, RANBP1, PPP2R2C, SPG20, COPS5, MAX, DKC1, EEF1E1, GLDC, MRPL19, TRAP1, IPO5, RASA1, NAE1, NPM1, YME1L1, CASP9, KPNA2, RPLP0, GPI, DHX9, SIRT3, CCT4, MYD88, KAT2A, NDUFS1, SSB, UTP6, CDH1, PDIA6, AMFR, HSPA9, CD14, HSP90AA1, MCL1, ITGB1, NOP56, GART, RAN, ASNS, RAF1, ALDH2, VDAC1, TAF10, ETFDH, GLUL, CAPRIN1, BCCIP, HIST2H2BE, RPL5, PARD3, LDHA, SIRT1, RUVBL2, NR3C1, ATP5B, RRM1, KARS, TLR1, CACYBP, SUPT3H, PPA1, BAK1, ALK, GARS, LIAS, DIABLO, DPY30, SUMO4, EIF4G2, EPRS |
| ***ITGB1*** | integrin, beta 1 (fibronectin receptor, beta polypeptide, antigen CD29 includes MDF2, MSK12) | Intellectual Disability, Mental Disorders, Brain Diseases, CNS Diseases, Neurologic Manifestations, Mental Disorders Diagnosed in Childhood, Neurobehavioral Manifestations | CRK, EGF, FABP3, ITGA4, LAMB1, LAMC3, PLAUR, PRKCB, RAC1, RELN, VEGFA, YWHAZ | NME1, COL1A1, CD82, RAP1B, FLT4, EGFR, VCAM1, ANGPT1, ITGB2, COL3A1, PRKCE, CD46, HSPD1, PTK2, FN1, SRC, MYC, VLDLR, COL4A4, CSF2RB, TGFBI, ITGA3, CSK, IGF1R, CD44, VEGFC, ITGAM, COL1A2, MAP4K4, ACTB, GNB2L1, TGOLN2, LAMA1, COL4A3, JAM2, VASP, THBS1, CD81, VCAN, EPS8, FLNB, ANGPTL4, FGB, LAMB2, CD36, PTP4A3, ITGB1BP1, ITGAL, MAX, FLNA, PLAU, ACTN1, ITGB4, LGALS1, CAV2, TNN, ACTN4, CD63, ITGA7, ILK, SDC2, DAG1, FBN1, FGG, CAV1, MKL1, RAP1A, VCP, TGM2, ITGA8, COL2A1, CD9, MMP2, VTN, PXN, TNR, KDR, SRF, PRKCA, ITGA5, TUBA1A, CD14, FLNC, ANKS1B, PTK2B, THBS4, CAV3, COL18A1, COL6A1, COL5A2, CANX, LAMA5, CHAD, F13A1, TNC, ITGB6, CSF2, JAM3, FGA, LGALS8, TRIB1, COL11A1, RAB25, THBS2, ADAM12, ZBTB17, COL4A1, CSF2RA, SPP1, BCAR1, ITGB8, RPS6KB1, IBSP, TIMP2, PRKCG, ITGA9, ITGA2, CSPG4, ACTG1, COL4A5, NF2, PRKAR1A, MDK, LAMC1, ITGAE, ITGA1, COL6A3, COL6A2, LRP8, HSPG2, TEK, LAMA2 |
| ***KIF1B*** | kinesin family member 1B | Intellectual Disability, Mental Disorders, Brain Diseases, CNS Diseases, Neurologic Manifestations, Nervous System Diseases, Neurodegenerative Diseases, Mental Disorders Diagnosed in Childhood, Neurobehavioral Manifestations |  | DLG1, BRCA1, MAGI1, CPEB1 |
| ***LAMP2*** | lysosomal-associated membrane protein 2 | Intellectual Disability, Mental Disorders, Brain Diseases, CNS Diseases, Neurologic Manifestations, Neurodegenerative Diseases, Mental Disorders Diagnosed in Childhood, Neurobehavioral Manifestations, X-linked Mental Retardation | ADRB2, ATRX, FMR1, SLC25A14, SLC9A6, UBE2A, CD99L2, PHF8 | PSMD10, SOD1, VBP1, ZMYM3, IDS, UBQLN2, REPS2, CTSA, PGK1, SNX12, ATP6AP2, MID2, ACSL4, RPGR, GK, BCAP31, JADE3, SLC35A2, PHF6, MTM1, OCRL, SH3KBP1, STAG2, CD63, UCHL1, FTSJ1, AP4M1, RPS6KA3, BRCC3, AIFM1, UXT, ATP7A, XIAP, SH3BGRL, PLP2, AMMECR1, RAB7A, ZNF41, ATP6AP1, MDM2, RP2, SELP, BRWD3, CASK, NKRF, RAP2C, RPL10, KRBOX4, WDR45, MTMR1 |
| ***LAMTOR3*** | late endosomal/lysosomal adaptor, MAPK and MTOR activator 3 | Nerve Tissue Neoplasms, Germ Cell and Embryonal Neoplasms |  | MAPK1, VAMP7, MAPK3, ENOPH1, CDKN2A, GMFB, TMCO1, MAP2K2, CHMP2B, MAP2K1, DPM1 |
| ***LGALSL*** | lectin, galactoside-binding-like | Autoimmune Diseases, Immune System Diseases, Congenital, Hereditary, and Neonatal Diseases and Abnormalities, Inborn Genetic Diseases |  |  |
| ***LYST*** | lysosomal trafficking regulator | Anxiety, Intellectual Disability, Mental Disorders, Neurologic Manifestations, Mental Disorders Diagnosed in Childhood, Neurobehavioral Manifestations | MED12, YWHAZ | YWHAQ, TNNI3, ATN1, CALM1, DGCR14, EFS, CELF4, CENPJ, HGS |
| ***MAP1LC3B*** | microtubule-associated protein 1 light chain 3 beta | Autistic disorder, Intellectual Disability, Mental Disorders, Neurologic Manifestations, Mental Disorders Diagnosed in Childhood, Neurobehavioral Manifestations | HTT | HDAC1, TBC1D25, MAP1A, ATG7, NBR1, MTMR14, UPF2, PINK1, SIRT1, MAP1B, TBK1, SNCA, SQSTM1, TBC1D5, OPTN |
| ***NFE2L2*** | nuclear factor (erythroid-derived 2)-like 2 | Mental Disorders, Brain Diseases, Central Nervous System Diseases, Nervous System Diseases | CREBBP | EIF2AK2, RARA, EIF2AK3, CASP3, GSK3B, UBC, JUN, MYC, PRKCH, CASP1, KEAP1, YY1, BACH1, PSMB5, CHD6, ATF4, NCOR2, PRKCA, SMARCA4, NCOR1, DDIT3, CDKN1A, PRKCG, SQSTM1, EIF2AK4, PPARG, PRKCD |
| ***NUDT16*** | nudix (nucleoside diphosphate linked moiety X)-type motif 16 |  |  |  |
| ***OCIAD1*** | OCIA domain containing 1 | Endocrine System Diseases |  | UBC |
| ***PANK2*** | pantothenate kinase 2 | Mental Disorders, Obsessive-Compulsive Disorder, Speech Diseases, Neurodegenerative Diseases, Anxiety, CNS Diseases, Communication Disorders, Brain Diseases, Language Disorders, Neurologic Manifestations, Mental Disorders Diagnosed in Childhood, Neurobehavioral Manifestations |  |  |
| ***PCNP*** | PEST proteolytic signal containing nuclear protein |  | TOMM20 | NAP1L1, UHRF2, UBC, MORF4L1, MATR3, MMADHC, DPM1 |
| ***PDE4B*** | phosphodiesterase 4B, cAMP-specific | Autistic Disorder, Psychotic Affective Disorders, Bipolar Disorders, Mental Disorders, Depressive Disorder, Psychotic Disorders, Schizophrenia, CNS disorders, Neurologic Manifestations, Mood Disorders, Schizophrenia and Disorders with Psychotic Features | ADSL, GMPR | DCK, ADCY1, ENTPD4, NPR1, APRT, GNAZ, PDE4A, ADK, ITPA, PPP1R1B, NT5E, HPRT1, ADCY6, AMPD1, ADCY2, NT5C3A, ADCY7, GUCY2D, AMPD2, ADCY8, ADCY9, GUK1, PDE4D, ADCY3, NPR2, AK2, GNAS, NT5C2, PDE4C, ADCY10, ENTPD1, AK1, ADCY5 |
| ***PFDN5*** | prefoldin subunit 5 | Parathyroid Diseases, Endocrine System Diseases | GLRX3 | EDF1, NDUFB2, ELP3, VBP1, HDAC1, RPL11, MYC, RPL24, HYPK, PSMC4, PSMD8, NDUFA4, RPL12, UQCRQ, PHF5A, PDCD5, EGR1, NAA38, NEDD8, CCT5, C11orf31, RPS19, TCP1, PARK7, SEC61G, IKBKG, TP73, CCT4, TRIM28, RPS9, SP1, PFDN2, SDHB, TUBA3C, BRMS1, BUD31, YEATS4, RPL29, SKP2, RPL30, POLR2G, HINT1, ITSN1, MRPL23, RPS24, TUBA1B, NDUFA1, NDUFS5, RPS6, RPL34, AIMP1, POLR2F |
| ***PHF20L1*** | PHD finger protein 20-like 1 |  |  | HIST1H3B, KAT8 |
| ***PNKD*** | paroxysmal nonkinesigenic dyskinesia | Brain Diseases, Central Nervous System Diseases, Cerebellar Diseases, Nervous System Diseases, Neurologic Manifestations, Neurodegenerative Diseases |  | ENO3 |
| ***PPM1B*** | protein phosphatase, Mg2+/Mn2+ dependent, 1B |  | MSN | CDK2, GRB2, VAV2, IKBKG, ARIH1, HERC5, SEC24B, IKBKB, MAP3K7, ISG15, PPP2CA, CDK6 |
| ***RAB24*** | RAB24, member RAS oncogene family |  |  | NSF |
| ***RAB2A*** | RAB2A, member RAS oncogene family | Mental Disorders |  | GAPDH, PRKCI, UBC |
| ***RBM25*** | RNA binding motif protein 25 |  |  | PNN, PPP2R2B, UBC, MGEA5, TCERG1, NIPBL, TPR, SON |
| ***RBM39*** | RNA binding motif protein 39 | Neoplasms, Carcinomas | TADA2A, SUMO1 | ESR1, PNN, UBC, JUN, NIPBL, SRRM2, U2AF2, TCF3, NR2C1, SRPK2, ESR2, MATR3, PGR, CTNNBL1 |
| ***RBMX*** | RNA binding motif protein, X-linked | Congenital, Hereditary, and Neonatal Diseases and Abnormalities , Nerve Tissue Neoplasms, Inborn Genetic Diseases | ATRX, FMR1, HSD17B10, MAGED1, MECP2, SLC25A14, SLC9A6, MED12, UBE2A, WNK3, FAM120C, CD99L2, UPF3B, PHF8 | PSMD10, VBP1, ZMYM3, IDS, UBC, UBQLN2, GPC3, PGK1, SNX12, ATP6AP2, LAMP2, MID2, ACSL4, RPGR, SCML1, JADE3, ABCB7, TAF1, PHF6, DKC1, OCRL, HDAC8, ELK1, STAG2, SCML2, FAM156A, FTSJ1, ITSN2, SLC25A5, PHKA1, MBTPS2,RPS6KA3, BRCC3, SORBS3, AIFM1, HPRT1, SLC9A7, UXT, ATP7A, PLP2, PHKA2, NHS, RBBP7, PDHA1, EBP, AMMECR1, ZNF41, KHDRBS3, IRAK1, PRPS1, BRWD3, APEX2, CASK, NKRF, RAP2C, RPL10, KRBOX4, WDR45, EMD, MTMR1, TIMM8A, PQBP1, CUL4B |
| ***RHEB*** | Ras homolog enriched in brain | Neoplasms | ATM, EIF4E, HRAS, TSC1, TSC2 | AKT1S1, PRKAG2, PRKAG3, RAP1A, PRKAA2, PLD1, MTOR, RAF1, RAB7A, BRAF, ATR, RPS6KB1, PRKAB2, EIF4EBP1 |
| ***RNF141*** | ring finger protein 141 |  |  |  |
| ***SEPT2*** | septin 2 | Leukemia Myeloid, Neoplasms | STX1A | PIK3R2, PIK3R1 , S100A4, SH3KBP1 , SEPT5, SEPT6 , SEPT7 , SNCA, ISG15 |
| ***SIRPA*** | signal-regulatory protein alpha | Nervous System Diseases, Brain Diseases, CNS Diseases | JAK2, VEGFA | VWF, GRB2, CD47, CGA, PTPN11, TYROBP, IL22RA2, NOL3, SYK, PTPN6, TREM2, TNFSF11, ARC |
| ***SLC44A2*** | solute carrier family 44 (choline transporter), member 2 | Nervous System Diseases, Neurologic Manifestations, Autoimmune Diseases |  | CHAT, ACHE |
| ***SORL1*** | sortilin-related receptor, L(DLR class) A repeats containing | Cognition Disorders, Mild Cognitive Impairment, Mental Disorders, Brain Diseases, CNS Diseases, Intellectual Disability, Neurologic Manifestations, Neurodegenerative Diseases, Mental Disorders Diagnosed in Childhood, Neurobehavioral Manifestations |  | GGA1 |
| ***SULF2*** | sulfatase 2 | Brain Disease, CNS Diseases, Nervous System Diseases |  |  |
| ***SUPT4H1*** | suppressor of Ty 4 homolog 1 (S. cerevisiae) |  |  | ELP3, POLR2A, ERCC6, PHF5A, GTF2H1 , PEX2, POLR2G, CDK7 , NELFE, POLR2F , PAF1, ERCC3, IKBKAP, PIN1,POLR2D, SUPT16H, NUFIP1, BUD31, NELFA, ELP4, NELFCD, ERCC2 |
| ***SVIL*** | supervillin | Vascular and Cardiovascular Diseases | ATRX, MYH9, MKL2 | ESR1, VCAM1, SH3PXD2A, SPTBN1, CENPF, LMNA, KIFC3, HSPA5, ACTB, DYNLT1, SPTAN1, FLNA, ACTN1, ACTN4, MPHOSPH9, TNFAIP1, CTTN, HSP90AA1, UXT, CAV3, HMMR, GOLGA2, VIM, HAX1, MMP25, MYLK, GPSM3, NR3C1, P2RX7, PPARG, KIF14, NEB |
| ***TCF4*** | transcription factor 4 | Autistic Disorder , Psychotic Affective Disorders, Bipolar Diseases, Developmental Disabilities, Cognition Disorders, Major Depressive Disorder, Language Development Disorders, Memory Disorders, Intellectual Disability, Psychotic Disorders, Seizures, Schizophrenia, Mental Disorders, Brain Diseases, CNS Diseases, Pervasive Child Developmental Disorders, Depressive Disorder, Epilepsy, Language Disorders, Neurologic Manifestations, Mental Disorders Diagnosed in Childhood, Neurobehavioral Manifestations, Mood Disorders, Schizophrenia and Disorders with Psychotic Features | MEN1, NEUROD1 | RNF138, ERBB2, CALM2, JUP, UBC, CALM1, PARP1, JUN, ID3, MYOD1, RUNX1T1, ASCL1, MSC, EP300, XRCC6, ID1, HBP1, BCL9, CTNNB1, ID4, TOP2A, ID2 |
| ***TDP2*** | tyrosyl-DNA phosphodiesterase 2 | Dyslexia, Mental Disorders, Brain Diseases, CNS Diseases, Language Disorders, Learning Disorders, Neurologic Manifestations, Neurodegenerative Diseases, Mental Disorders Diagnosed in Childhood, Neurobehavioral Manifestations | CD40LG, SUMO1 | UBC, TNFRSF1B, ERG, NFKBIA, CD164, UBE2I, ETS2, ETS1, TRAF3, TRAF5, RELA, NFKB1, FLI1, GABPA, CD40 |
| ***TIMP2*** | TIMP metallopeptidase inhibitor 2 | Alzheimer Disease, Autoimmune Diseases, Memory Disorders, Mental Disorders, Brain Disorders, CNS Diseases, Cerebellar Diseases, Mental Disorders Diagnosed in Childhood, Neurobehavioral Manifestations | MMP16 | ITGA3, MMP9, ITGA8, MMP2, ITGB1, MMP8, MMP14 |
| ***TM9SF3*** | transmembrane 9 superfamily member 3 |  |  |  |
| ***TNRC6A*** | trinucleotide repeat containing 6A | Autoimmune Diseases of the Nervous System, Nervous System Diseases, Systemic Lupus Erythematosus, Musculoskeletal Diseases |  | PABPC1, AGO2, UBR5 |
| ***TPM1*** | tropomyosin 1 (alpha) | Nervous System Diseases, Neuromuscular Diseases, Musculoskeletal Diseases, Congenital, Hereditary, and Neonatal Diseases and Abnormalities, Inborn Genetic Diseases, X-linked Genetic Diseases | DMD | EPB41, TNNI3, UBC, JUN, MYBPC3, MYL4, ACTB, MYH11, TNNT3, TCAP, LMOD1, TNNC2, MYL3, SORBS3, CNN1, TNNC2, MYL3, SORBS3, CNN1, TNNT1, VIM, TNNI2, MYLK, DES, PKD2, MYBPC1, MYH3, MYL2, ITGA1, MYL1, TPM2, SORBS1, NEB |
| ***TXN*** | thioredoxin | Cognition Disorders, Schizophrenia, Mental Disorders, Brain Diseases, CNS Diseases, Neurologic Manifestations, Sleep Disorders, Neurodegenerative Diseases, Schizophrenia and Disorders with Psychotic Features | MSRA | COL1A1, GAPDH, NDUFB3, TP53, EGFR, HMGB1, UBC, RPL11, PTPN1, APEX1, COPS5, TXNIP, RRM2B, PRDX2, TRADD, MYD88, TNFRSF1A, VIM, NFKB1, GUK1, MAP3K5, NR3C1, RRM1, HINT1, NLRP3, GFER, PRDX1, AK1, CKS2 |
| ***UBE2D3*** | ubiquitin-conjugating enzyme E2D 3 | Leukemia Myeloid, Nutrition Disorders | NEDD4, UBE3A, TRIM32 | TP53, DNAJA2, UBA1, UBC, BRCA1, DNAJB1, CSNK1A1, MID2, DDX58, NFKBIA, STUB1, BIRC2, HIST2H2AC, HDAC6, DNAJB2, DNAJC5, IKBKG, RNF5, RNF11, BARD1, XIAP, RBCK1, BTRC, RELA, TRIM31, NFKB1, BMI1, MDM2, TRIM37, SAT1, ISG15, UBE4B |
| ***UPF2*** | UPF2 regulator of nonsense transcripts homolog (yeast) | Autistic Disorder | MAP1LC3B, SMG6, UPF3B | TP63, XRN1, RPL11, UPF1, RPL12, RPS19, MAP1A, RPLP0, SKIV2L2, RPS9, MAP1LC3B2, SMG1, RPL29, RPL3, RPL5, NOB1, RPL30, UPF3A, RPS24, RPL37, EIF4G1, RPS17, RPS6, RPL34 |
| ***VMP1*** | vacuole membrane protein 1 | Neurodegenerative Diseases, Nervous System Diseases, Immune System Diseases |  | UBC , HGS, TJP1 |
| ***VPS29*** | vacuolar protein sorting 29 homolog (S. cerevisiae) | Nervous System Diseases, Neurologic Manifestations, Neurodegenerative Diseases, Peripheral Nervous System Diseases, Congenital, Hereditary, and Neonatal Diseases and Abnormalities, Inborn Genetic Diseases, Neuromuscular Diseases, Polyneuropathies, Paralysis |  |  |
| ***WIPF1*** | WAS/WASL interacting protein family, member 1 | Brain Diseases, CNS Diseases, Colonic Diseases, Gastrointestinal Diseases, Immune System Diseases, Nervous System Diseases, Inflammatory Bowel Diseases, , Inborn Genetic Diseases, X-linked Genetic Diseases, Congenital, Hereditary, and Neonatal Diseases and Abnormalities |  | GRB2, ACTC1, BAIAP2, WAS, NCK2, SH3KBP1, ITSN2, CTTN, ZAP70, SYK, EVI2B |
| ***WLS*** | wntless Wnt ligand secretion mediator | Musculoskeletal Diseases, Nerve Tissue Neoplasms |  | UBC, OPRM1 |
| ***ZNF644*** | zinc finger protein 644 | Eye Diseases | ATRX | SMC3, NIPBL |

**Supplementary Table S2**. Root 66 genes description, interesting disease networks they are involved in and interaction with ASD candidates and/or genes associated to neurological conditions.

| **Groups K=14** | **Biosets** | **Mean Jaccard Coefficient** |
| --- | --- | --- |
| **1** | 18123_Asperger_GPL570, 18123_autism_GPL570, 18123_PDD_GPL570, 38322_aut_cb, 28475_aut_FFPE_DASL | 0.6174925 |
| **2** | 18123.PB.Asperger.GPL6244, 18123.PB.aut.GPL6244, 18123.PDD.GPL6244 | 0.8775857 |
| **3** | 25507.PBL.aut | 0.6427500 |
| **4** | 28475.Brain.FFPE.invitro | 0.6560000 |
| **5** | 28475.Brain.Frozen.DASL, | 0.6385000 |
| **6** | 28475.Brain.Frozen.Invitro, | 0.6580000 |
| **7** | 28521.cb.aut | 0.6260000 |
| **8** | 28521.cortex.aut, 28521.gyrus.aut | 0.8620167 |
| **9** | 37772.LCL.aut | 0.6435000 |
| **10** | 38322.lobe.aut | 0.6391500 |
| **11** | 39447.IPSC.aut, 39447.NPC.aut, 39447.Fibroblast.aut, 39447.Neurons.aut | 0.6362631 |
| **12** | 6575.WB.Autism, 6575.WB.AutR | 0.8155786 |
| **13** | 6575.WB.AutNR, 6575.WB.MM.DD | 0.7130476 |
| **14** | 7329_aut_FX, 7329_aut_dup, | 0.8603000 |

**Supplementary Table S3**. Biosets integrating each group generated by the bootstrap and their Mean Jaccard Coefficient value. All the clusters are above the established margin that considers a cluster indicator of a pattern in the data.

**Supplementary Figure Legends**

**Supplementary Figure S1.** Number of differentially expressed genes/probes and tissue type for each clustered experiment.

**Supplementary Figure S2**. First two Multidimensional Scaling (MDS) dimensions of our dataset generated by MDS on a dissimilarity matrix using Jaccard Coefficient when k=14. The biosets conforming Root 66 are clustered together in group 1. The subsets that are plotted closer in the graph are the corresponding to GSE7329 and GSE3944 and GSE28475 experiments, groups 14, 11 and 5 respectively.

**Supplementary Figure S3**. Neuroendocrine and Normal Development network. Root 66 genes are highlighted in purple; interactions are represented in blue; it has a score of 27.

**Supplementary Figure S4**. Neurodegeneration Network. Root 66 genes are highlighted in purple; interactions are represented in blue; its score is 24.

**Supplementary Figure S5**. Neurodegeneration and tumor network. Root 66 genes are highlighted in purple; interactions are represented in blue. Network score is 22.

**Supplementary Figure S6**. Distribution representing the number of Root 66 genes found in each pairwise bioset intersection.
