# Supplementary figures and images for: A common molecular signature in ASD gene expression: following Root 66 to autism
Source: Transl Psychiatry. 2016 Jan 5;6(1):e705–. doi: 10.1038/tp.2015.112 (PMC5068868; doi:10.1038/tp.2015.112)

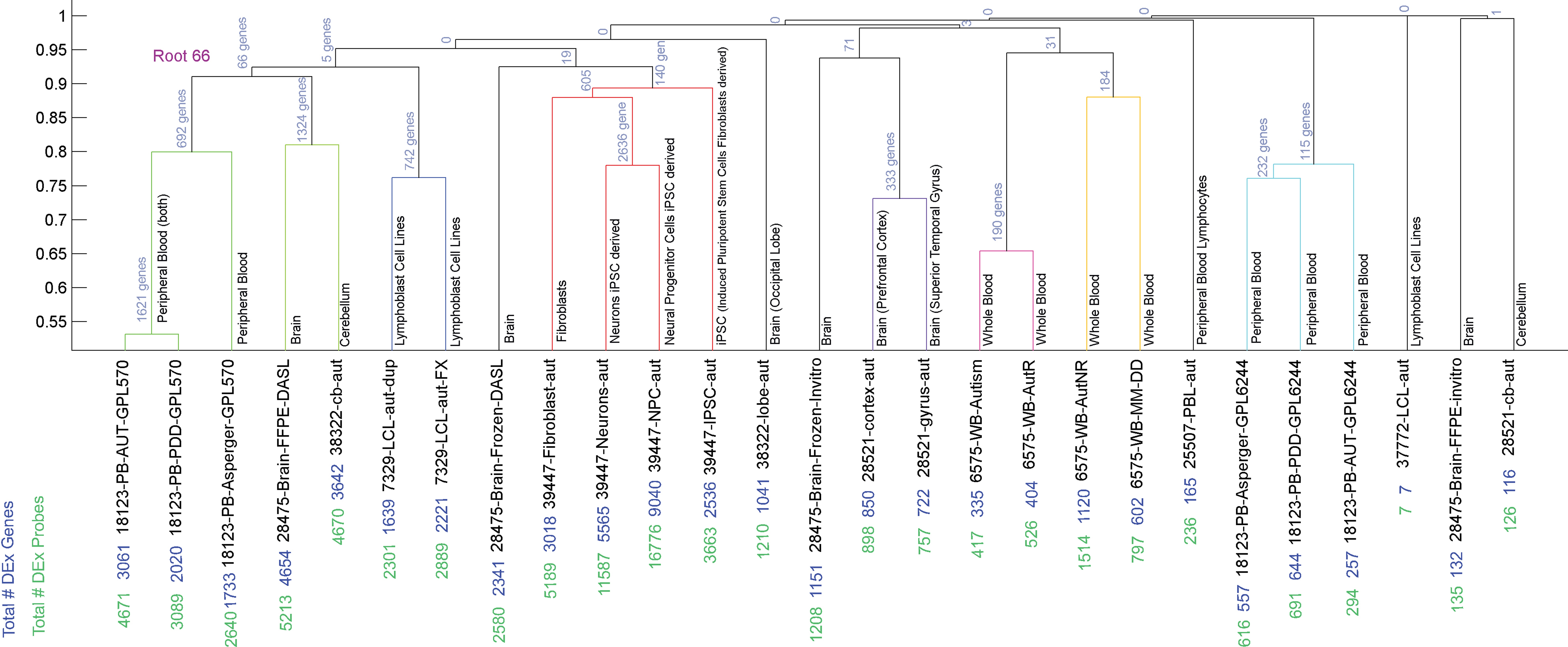

Supplement: Supplementary Figure 1 [file tp2015112x2.tif]

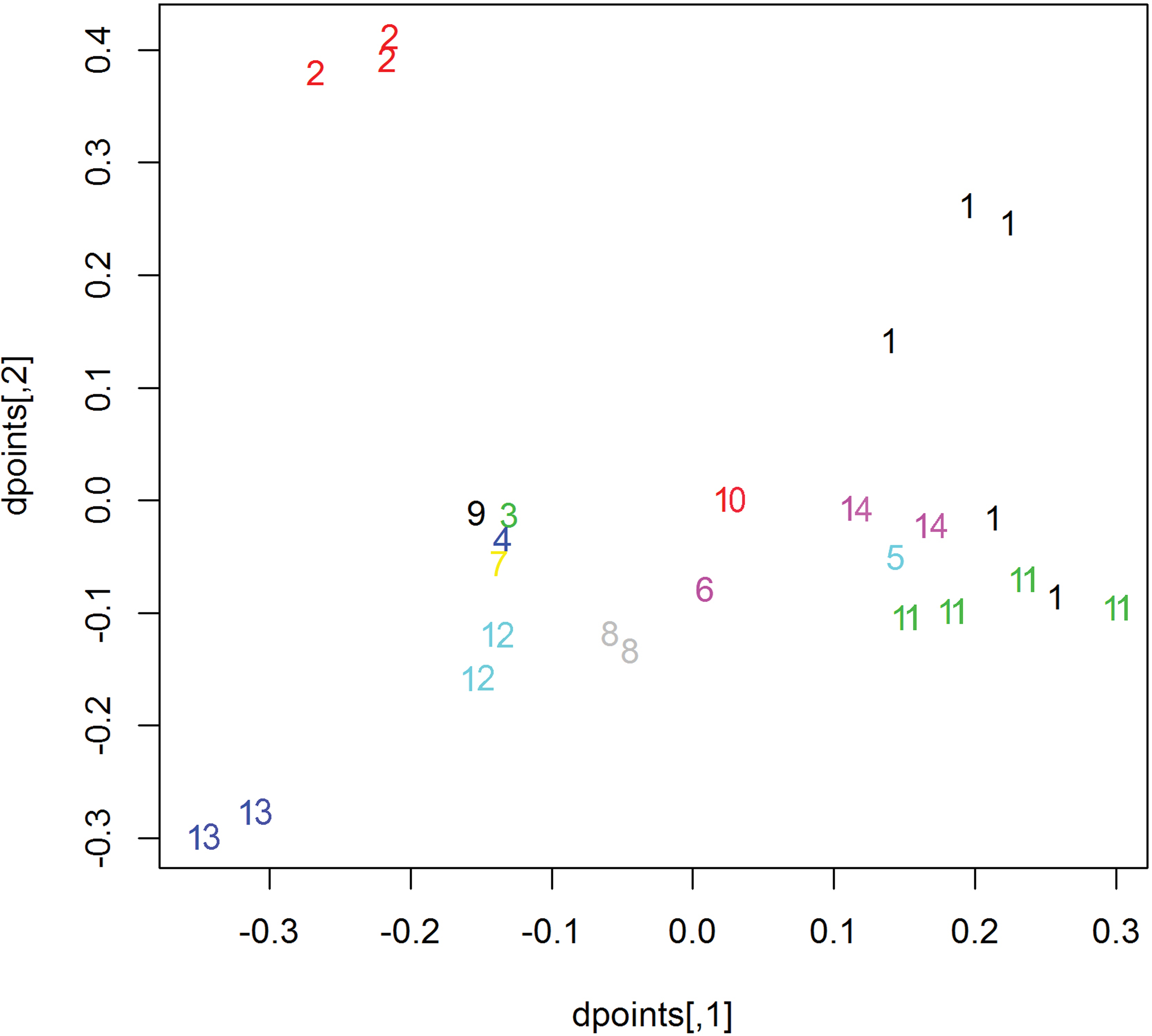

Supplement: Supplementary Figure 2 [file tp2015112x3.tif]

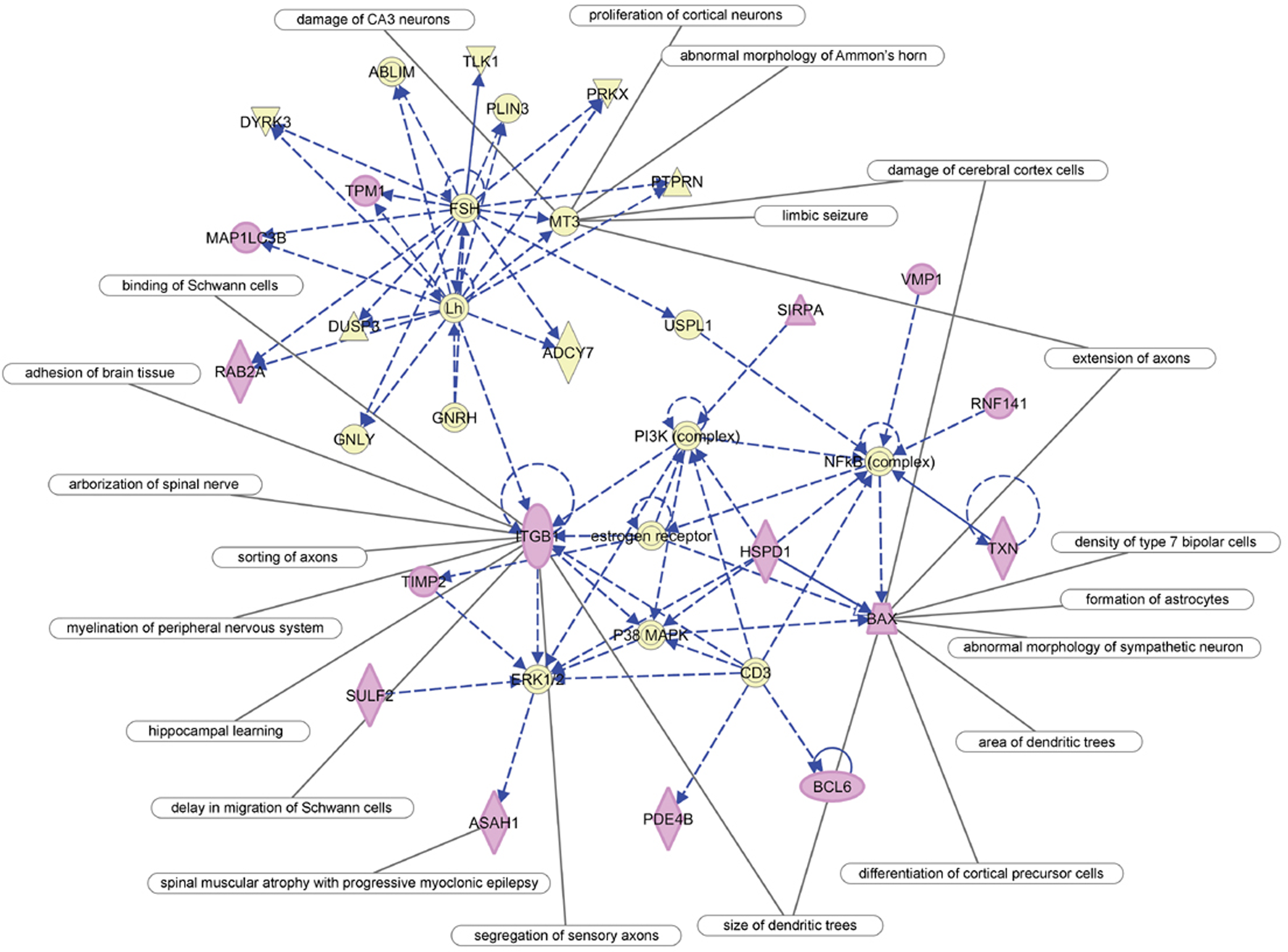

Supplement: Supplementary Figure 3 [file tp2015112x4.tif]

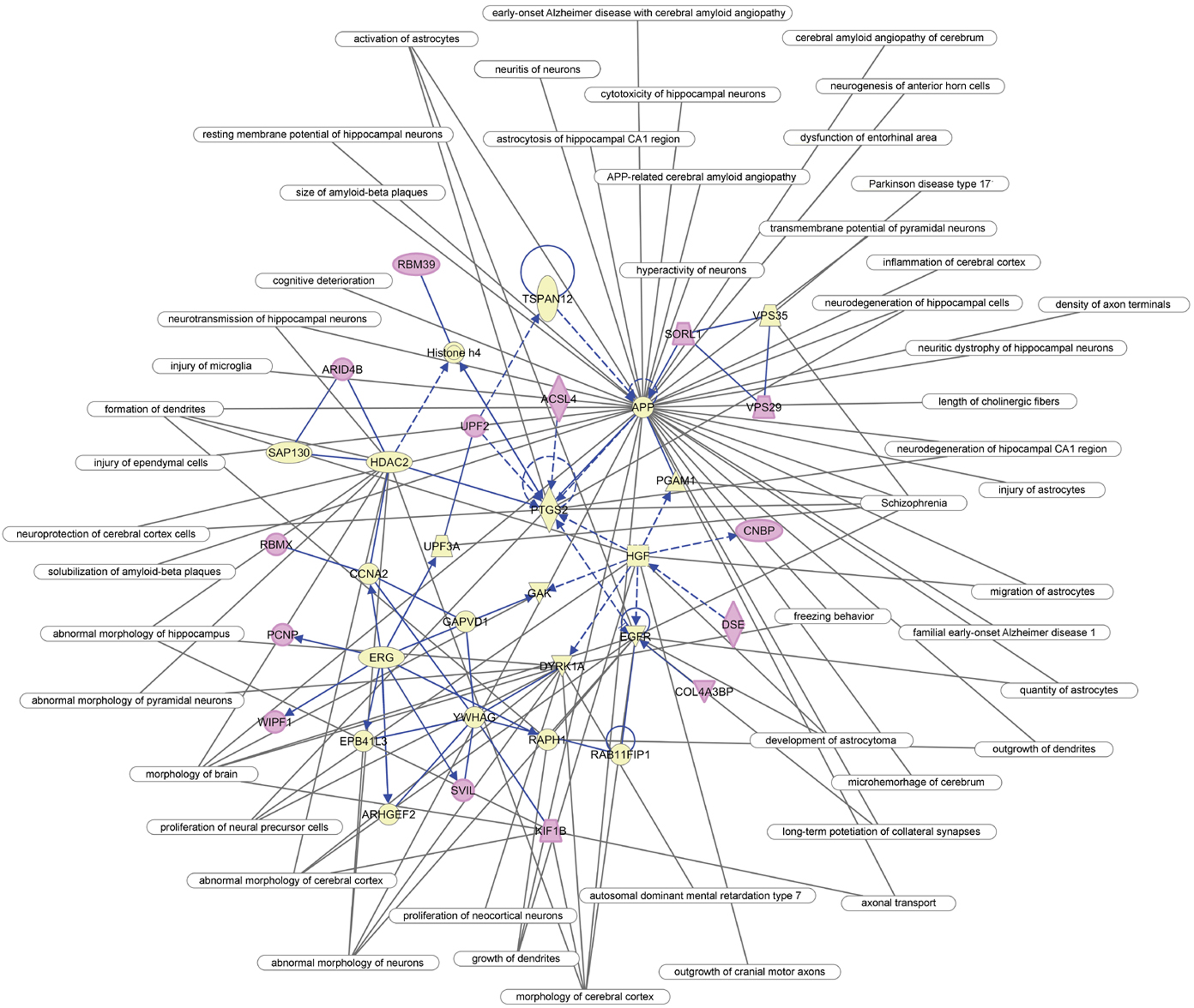

Supplement: Supplementary Figure 4 [file tp2015112x5.tif]

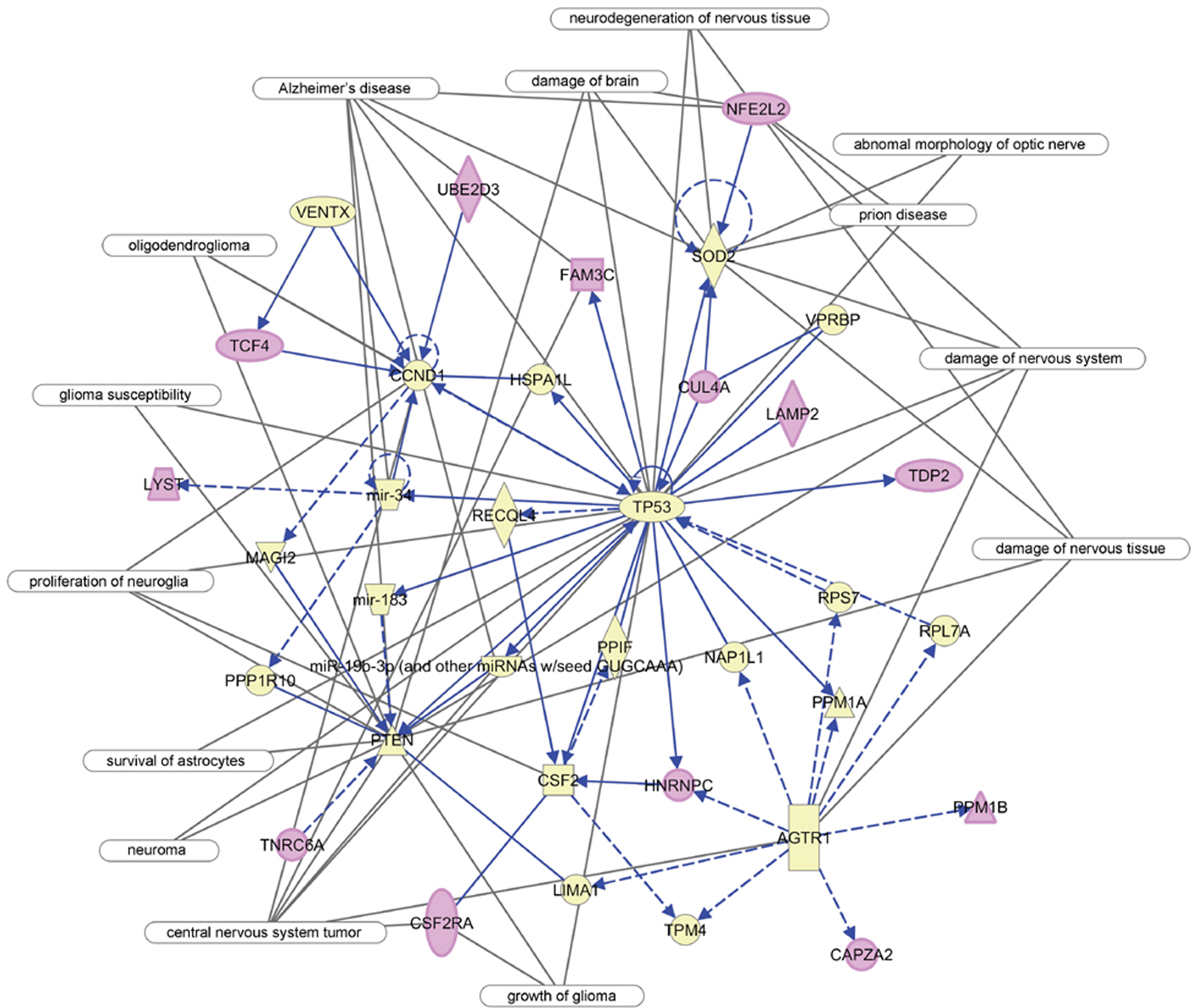

Supplement: Supplementary Figure 5 [file tp2015112x6.tif]

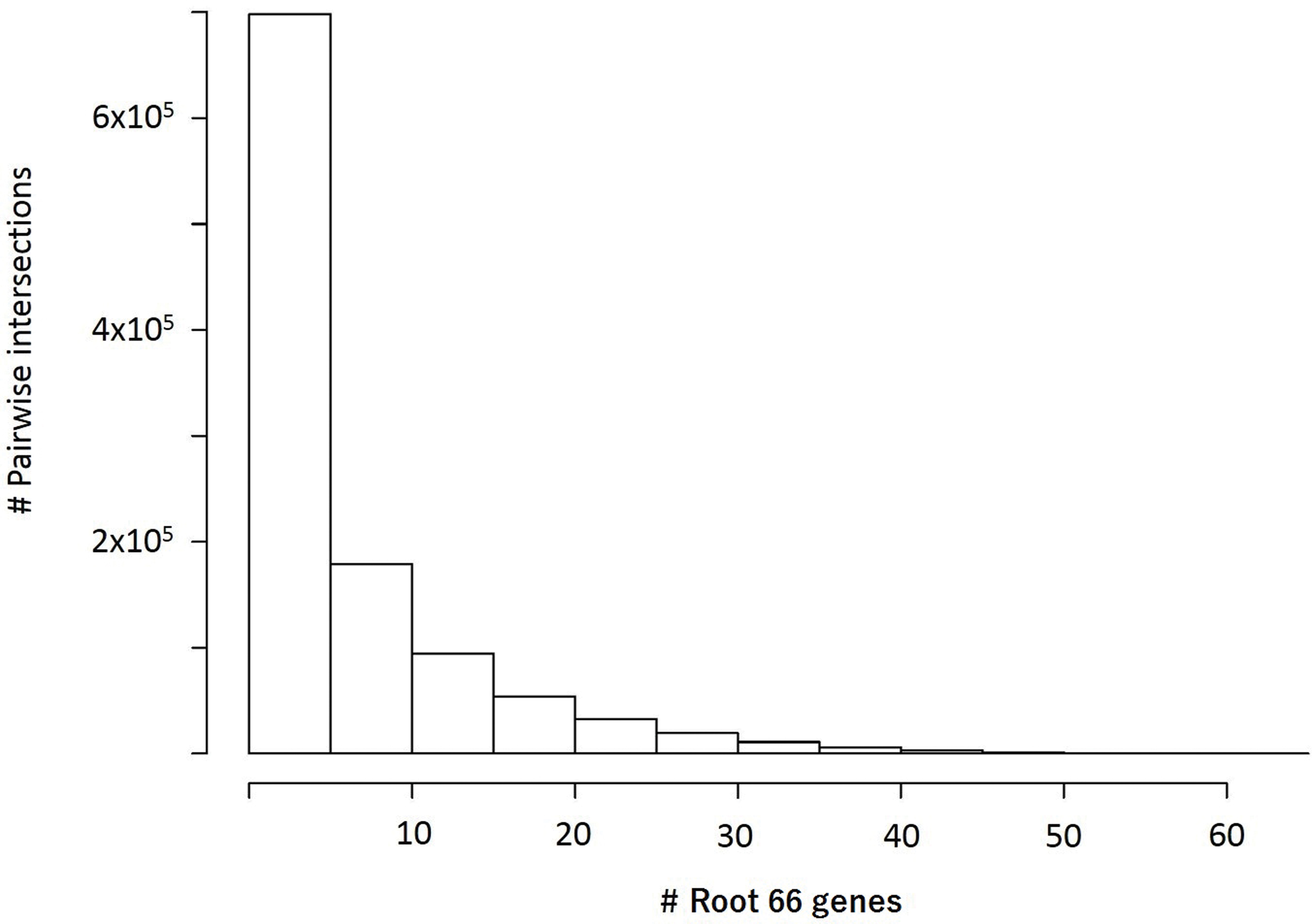

Supplement: Supplementary Figure 6 [file tp2015112x7.tif]
